# PGC‐1α Activation by Polyphenols: A Pathway to Thermogenesis

**DOI:** 10.1002/mnfr.70072

**Published:** 2025-04-29

**Authors:** Nicholas Vannuchi, Luciana Pisani

**Affiliations:** ^1^ Departamento de Biociências, Laboratório de Nutrição e Fisiologia Endócrina (LaNFE) Instituto de Saúde e Sociedade Universidade Federal de São Paulo Santos São Paulo Brazil

**Keywords:** adipose tissue, beige adipose tissue, brown adipose tissue, lipid metabolism, phenolics, browning

## Abstract

This review investigates the role of polyphenols, abundant natural compounds found in food, to influence the metabolic pathways involved in the thermogenesis and browning of white adipose tissue (WAT). Numerous proteins demonstrate altered expression patterns following prolonged polyphenol consumption, with peroxisome proliferator‐activated receptor gamma coactivator 1‐alpha (PGC‐1α) recognized as a key regulator, contributing to increased thermogenicity of adipose tissues. Polyphenols may enhance PGC‐1α activity, stimulating WAT browning, and elevating brown adipose tissue (BAT) thermogenesis. Various classes of polyphenols are explored, along with extensive protein signaling and the physiological implications of these findings. A comprehensive understanding of the myriad proteins and pathways implicated in browning studies can provide readers with a broader perspective on the modulated response of adipose tissue to polyphenols and guide them to innovative therapeutic strategies for lipid metabolism, obesity, and associated metabolic disorders.

## Graphical abstract

Polyphenols, derived from dietary sources such as berries, undergo digestion and interact with gut microbiota, leading to their metabolization in the liver. This process influences bile acid composition, which in turn activates PGC1α, a key regulator of mitochondrial biogenesis and thermogenesis. PGC1α activation promotes adipose tissue browning, enhancing energy expenditure and metabolic health. This pathway highlights the role of polyphenols in improving mitochondrial function and thermogenic responses.

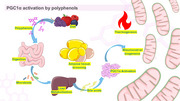

## 1 Introduction

Adipose tissue, once considered a mere energy storage depot, has emerged as a highly dynamic endocrine organ that plays a pivotal role in regulating whole‐body metabolism [1]. Among its various functions, the capacity of white adipose tissue (WAT) to undergo “browning”—a process characterized by the acquisition of brown‐like adipocyte features—has garnered significant attention [2]. This phenotypic switch is accompanied by increased energy expenditure and enhanced insulin sensitivity, which correlate with better metabolic status overall.

Brown adipose tissue (BAT) is strategically distributed in the body, particularly in the supraclavicular, axillary, cervical, paraspinal, mediastinal, and abdominal regions in humans, and the intrascapular region in mice. While abundant in newborns and infants, BAT mass decreases as we age [3]. UCP1 is located in the mitochondrial inner membrane and stimulates thermogenesis by dissipating the protonmotive force maintained by the electron transport chain (responsible for pumping electrons out), otherwise leaking protons in the inner membrane. In response, to keep homeostasis, an increased rate of substrate influx is driven to mitochondria consuming therefore more energy [4]. Despite their functional similarities, brown and beige adipose tissues arise from distinct embryonic lineages. Beige adipose tissue, shares a closer developmental origin with white fat, while brown adipocytes originate from progenitor cells common to muscle cells (myocytes) [5], and are regulated by a different set of chromosomes and mechanisms [6, 7].

Given that obesity arises from a chronic imbalance between energy intake and expenditure, interventions that augment energy expenditure are considered a promising therapeutic strategy. Recent investigations suggest that upregulating thermogenic activity within adipose tissues presents a potential therapeutic avenue for treating obesity in both human and murine models [8, 9, 10, 11].

Polyphenols, a diverse group of naturally occurring compounds found in plant‐based foods, have been extensively studied for their potential health benefits, including their antioxidant, anti‐inflammatory, and anti‐carcinogenic properties [12, 13, 14, 15]. Recent research has highlighted the intriguing possibility that polyphenols may also modulate adipose tissue function, particularly by promoting browning. The peroxisome proliferator‐activated receptor gamma coactivator 1‐alpha (PGC‐1α) is a master regulator of mitochondrial biogenesis and function. It plays a central role in the browning process by coordinating gene expression in thermogenesis. Several studies have demonstrated that polyphenols can activate PGC‐1α, stimulating WAT's browning and enhancing brown adipose tissue (BAT) non‐shivering thermogenesis [16, 17].

This review will delve into the molecular mechanisms underlying the effects of polyphenols on adipose tissue browning with a particular focus on the role of PGC‐1α. We will discuss the various classes of polyphenols that have been shown to promote browning, the potential signaling pathways involved, and the physiological implications of these findings. Understanding the mechanisms by which polyphenols modulate adipose tissue browning may provide novel therapeutic strategies for treating obesity and related metabolic disorders.

## 2 VosViewer Analysis

**FIGURE 1:**
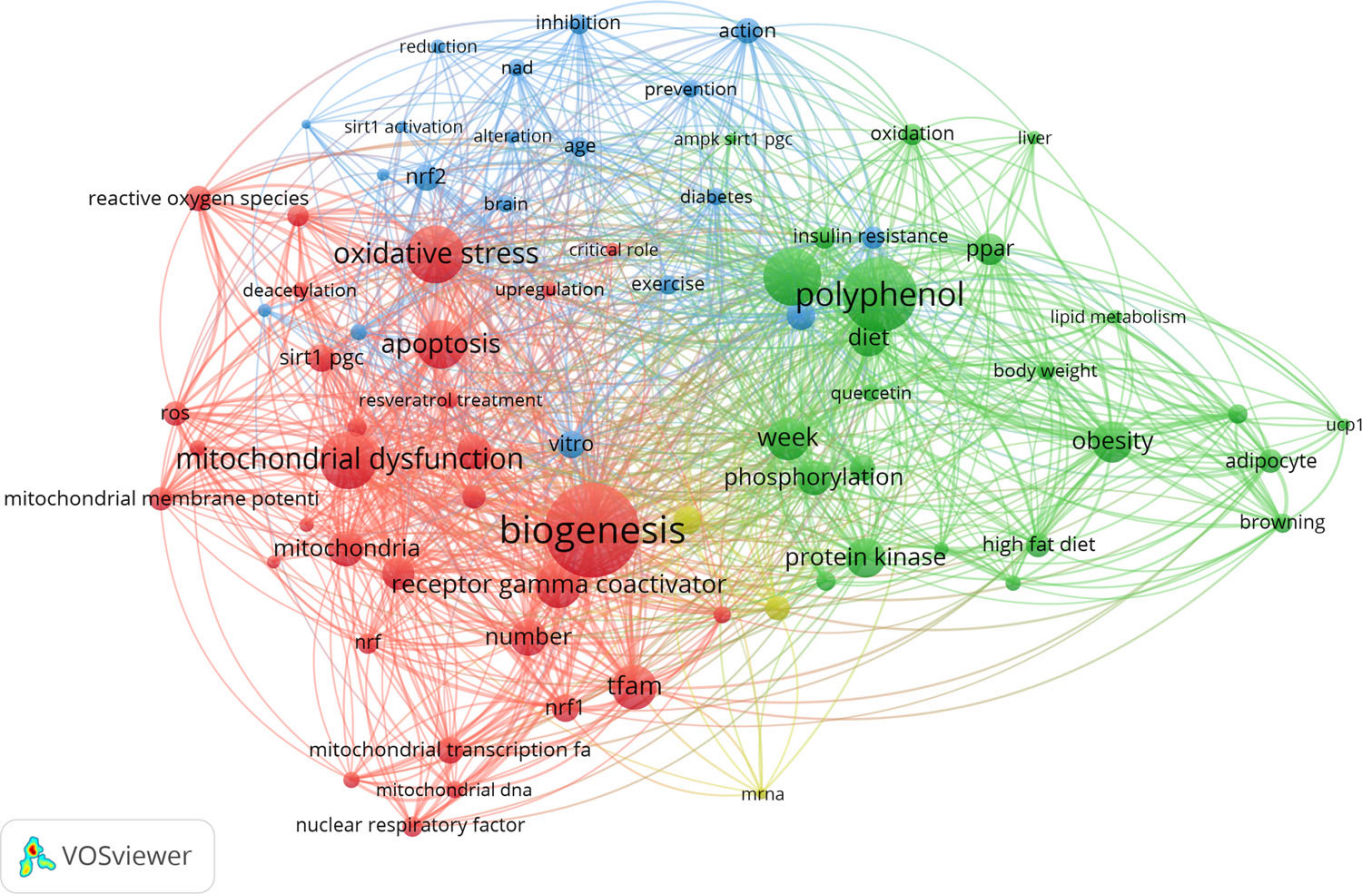
VosVIEWER image using the term “((PGC‐1α) OR (PGC‐1alpha)) AND (POLYPHENOL)” in PubMed database from 2006 to 2024. **Red Cluster**: Related to mitochondrial function and regulation; this cluster focuses on the processes within mitochondria, including energy processes. Terms like SIRT1, NRF1, UCP1, and TFAM indicate a focus on mitochondrial biogenesis, mitochondrial gene expression regulation, and metabolism regulation. Terms such as “browning,” “lipid metabolism,” and “insulin resistance” link mitochondria to know energy management processes well. **Green Cluster**: Related to polyphenols and metabolism; this cluster explores the beneficial effects of polyphenol consumption. In addition to the terms common to the red cluster, such as SIRT1 and PPAR, we find terms like “AMPK,” “diet,” “body weight,” and “obesity,” indicating an interest in how polyphenols can influence energy regulation, weight control, and the prevention of metabolic diseases. **Blue Cluster**: Related to mitochondrial dysfunction and associated diseases; this cluster focuses on the negative effects of mitochondrial dysfunction and strategies to prevent them, including nutrition. Terms such as “degenerative diseases” and “exercise” suggest an interest in how mitochondrial dysfunction can contribute to the development of diseases and how lifestyle can help prevent or reverse these effects.

By searching the PubMed database for the terms PGC‐1α (or PGC‐1alpha) and polyphenol, we identified 236 relevant articles published between 2006 and 2024. To visualize the interconnections among the words found in the titles and abstracts of these articles, we employed VosViewer software to create a bibliometric network (Figure 1). This network represents the most frequently occurring terms, clustered by color to indicate their proximity with other areas of interest in the field. Our analysis revealed the following three primary clusters: terms related to mitochondrial function and apoptosis (red), polyphenols and obesity (green), and other degenerative diseases and prevention (blue).

## 3 STRING Generated Network of PGC‐1α Protein Interactions

**FIGURE 2:**
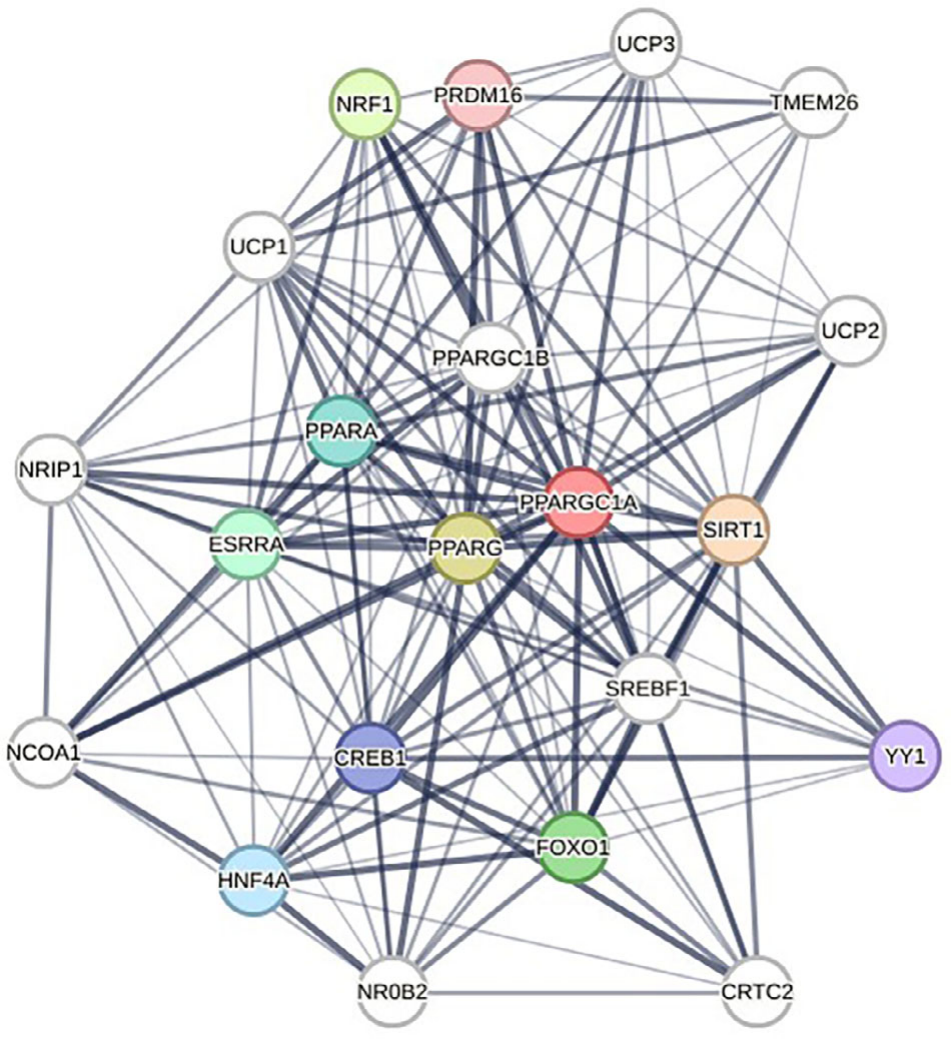
STRING map, with predicted protein–protein interactions, focusing PGC‐1α protein for *Homo sapiens*. The interactions may include both direct (physical) and indirect (functional) associations from scientific databases. CREB1 (cAMP response element‐binding protein 1); ESRRA (estrogen receptor‐related receptor alpha); FOXO1 (Forkhead box O1); HNF4A (hepatocyte nuclear factor 4 alpha); NCOA1 (nuclear receptor coactivator 1); NR0B2 (nuclear receptor subfamily 0 group B member 2); NRIP1 (nuclear receptor interacting protein 1); PPAR (peroxisome proliferator‐activated receptor); PPARA (peroxisome proliferator‐activated receptor alpha); PPARG (peroxisome proliferator‐activated receptor gamma); PPARGC1A (peroxisome proliferator‐activated receptor gamma coactivator 1 alpha); PPARGC1B (peroxisome proliferator‐activated receptor gamma coactivator 1 beta); PRDM16 (PR domain containing 16); SIRT1 (Sirtuin 1); SREBF1 (sterol regulatory element‐binding transcription factor 1); TMEM26 (transmembrane protein 26); UCP1 (uncoupling protein 1); UCP2 (uncoupling protein 2); UCP3 (uncoupling protein 3); YY1 (Yin Yang 1); NRF1 (nuclear respiratory factor 1).

String (https://string‐db.org/) serves as a resource for investigating protein–protein interactions, integrating both experimentally determined and computationally predicted associations to generate functional association networks. Below, an association grid was generated with the first level of interactions of PGC‐1α protein (Figure 2) for humans. As we can see in these networks, available data correlate PPARGC1α (the gene that codifies PGC1α); PPARα (peroxisome proliferator‐activated receptor alpha); PPARG (peroxisome proliferator‐activated receptor gamma), and SIRT1 (Sirtuin 1) are the most common protein associated to PGC‐1α in function, closely working together to maintain mitochondrial and energetical function of the body. Among the second level (white circles), UCP isoforms and SREBF1 (Sterol regulatory element‐binding transcription factor 1) are the most commonly cited in polyphenol studies. Increased UCP levels in the adipose cells are associated with “browning,” an aspect acquired by the adipose tissue and brown adipose tissue due large number of mitochondria that increase in number, size, and usually lamellae.

## 4 Enhancing White Adipose Tissue Browning and Brown Adipose Tissue Activation

**FIGURE 3:**
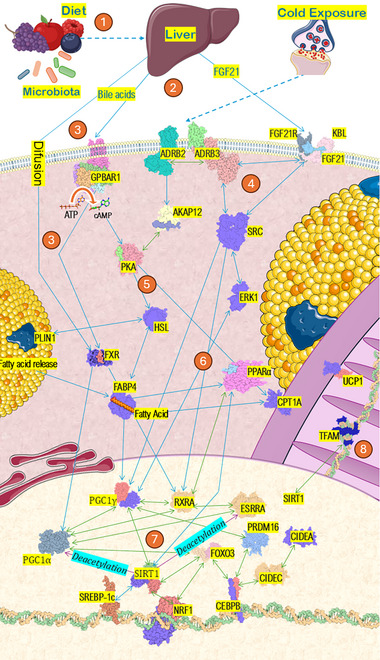
This diagram depicts the pathways and interactions between various proteins involved in liver function, particularly focusing on how polyphenol consumption triggers bile acid and FGF21 signaling. ADBR2 indicates Beta‐2 adrenergic receptor; ADBR3, Beta‐3 adrenergic receptor; AKAP12, A‐kinase anchoring protein 12; CEBPB, CCAAT/enhancer‐binding protein beta; CIDEA, cell death‐inducing DFFA‐like effector A; CIDEC, cell death‐inducing DFFA‐like effector C; ERK, extracellular signal‐regulated kinase; ESRRA, estrogen‐related receptor alpha; FABP4, fatty acid‐binding protein 4; FGF21, fibroblast growth factor 21; FGF21R, fibroblast growth factor 21 receptor; FOXO3, Forkhead box protein O3; FXR, Farnesoid X receptor; HSL, hormone‐sensitive lipase; NRF1, nuclear respiratory factor 1; PLIN1, Perilipin‐1; PKA, protein kinase A; PPAR, peroxisome proliferator‐activated receptor; PRDM16, PR domain containing 16; PGC1A, peroxisome proliferator‐activated receptor gamma coactivator 1‐alpha; PPARG, peroxisome proliferator‐activated receptor gamma; RXARA, Retinoid X receptor alpha; SIRT1, Sirtuin‐1; SRC, proto‐oncogene c‐Src; TFAM, mitochondrial transcription factor A; UCP1, uncoupling protein 1. (1) The body metabolizes polyphenols through microbiota interaction, creating smaller metabolites. (2) The liver is stimulated by these metabolites to produce bile acids, SCFA, and FGF21. (3) Bile acids activate GPBAR1 and FXR generating a signal that leads to PGC1α activation. (4) FGF21 binds its receptor and interacts with adrenergic receptors, which are also involved in cold‐induced thermogenesis. (5) The FGF21 signal is relayed by kinase proteins such as SRC and PKA and contributes to thermogenesis by activating PPAR, RXRA, and PGC1γ. (6) PKA also phosphorylates HSL, PLIM, and CPT1A, which improves the lipid mobilization to further fuel the thermogenic process. (7) PGC1α is the main co‐factor that interacts with many other co‐factors in the nucleus that coordinate enhanced mitochondrial biogenesis‐related genes, both in the nucleus and mDNA genome through Mitochondrial Transcription Factor A (TFAM, 8). The proteins were visualized using ChimeraX and PyMOL, with connections established based on findings from relevant research papers and data from STRING‐db.org protein networks. Green arrows: complex formations. Blue arrows: molecule interactions.

The activation of BAT and browning of WAT are multifaceted processes, triggered by multiple metabolic pathways. The intrinsic complexity of cellular tracks, characterized by high sensitivity and responsiveness to multiple stimuli, obstructs the delineation of a main, single signaling pathway. With this in mind, PGC1α is often called the “master regulator of the browning process,” serving as a central receptor of many external and internal signals. The protein is known to interact with at least 162 molecules [18], including many downstream and upstream small proteins. Although PGC‐1α is not strictly necessary for browning to occur, cells deficient in either PGC‐1α or isoform PGC‐1β exhibit impaired mitochondrial biogenesis and reduced expression of most related proteins [19]. The protein interactions supporting mitochondrial biogenesis, enhanced lipid metabolism, and activating non‐shivering thermogenesis are described below and illustrated in Figure 3.

Thermogenesis is initiated physiologically by cold sensation, exercise, or dietary intake [20, 21], which stimulates the release of norepinephrine from sympathetic nerves to activate adrenergic receptors (ADBR) in adipose tissue [22]. Additionally, AMPKα2 is known to upregulate UCP1 expression through PGC1α to enhance white adipose tissue (WAT) browning and brown adipose tissue (BAT) activation [23, 24].

## 5 Reaching PGC1α Activation by Polyphenol Consumption

### 5.1 Oral Consumption

Polyphenols undergo substantial metabolic transformations during digestion and subsequent intracellular signaling before exerting their thermogenic effects in adipose tissues. These effects are primarily attributed to smaller molecules, such as caffeic and protocatechuic acids, bile acids (BA), and short‐chain fat acids (SCFAs), derived from the biotransformation mediated by digestive enzymes and the gut microbiota (GM) [12]. The GM plays a pivotal role in this process, specifically the bacteria, as evidenced by the attenuation of polyphenol extract‐induced thermogenesis following antibiotic administration [25]. These smaller molecules reach the liver and promote the release of at least fibroblast growth factor 15 and 21 (FGF15/21) [26, 27] and bile acids [28], which then circulate in the bloodstream and reach other tissues.

FGF21 binds to its specific receptor on the surface of adipose cells (FGF21R), a fusion in which β‐klotho (KLB) is also required [29]. This initiates a complex signaling cascade that leads to improved lipid metabolism and thermogenesis stimulation [30]. FGF21R also acts together with β‐adrenergic type 3 (ADRB3) to induce thermogenesis, as FGF21‐KO mice are unresponsive to browning via adrenergic agonists [31]. On the other hand, FGF21 is not essential for the browning of inguinal white adipose tissue (iWAT) in mice exposed to cold [32], and FGF21 production stimulated by fructose consumption does not seem to activate thermogenesis in Japanese women alone [33]. Interestingly, in humans, relatively less ADBR3 in adipocytes is found [34]. Strong evidence links human thermogenesis stimulation by noradrenergic agonists to isoform ADBR2, instead of ADBR3, the latter traditionally associated with noradrenergic response in mice and rats [35]. This is an important difference that thermogenesis researchers must keep in mind when evaluating β‐adrenergic stimulation and thermogenesis in rodents.

Conversely, FGF21 appears to be necessary for phenolic compounds to exert their beneficial effects. In an intervention with curcumin and resveratrol, male mice lacking FGF21 (FGF21‐KO) did not exhibit the same reductions in total glycerides and improvements in fat tolerance as those with intact FGF21 function [31]. Nevertheless, for diet‐induced obese mice, FGF21 plays a critical role in maintaining the body homeostasis in mild cold conditions [36], which implies that FGF21 might be more involved in lipid and calcium metabolism, and just indirectly involved with fueling UCP1 thermogenesis. Since the adipocyte can also produce this factor in an autocrine fashion [37, 38], FGF21 role may match that of a signal amplification, where adipocytes stimulate the whole tissue to induce browning in response to the liver's initial signaling [30, 39].

From the bile acids perspective, primary BAs synthesized in the liver are conjugated with taurine in mice and glycine in humans [40]. Salt hydrolases deconjugate these amino acids from the primary BAs, allowing for bacterial biotransformation. This process involves reactions like 7α/β‐dehydroxylation, dehydrogenation, and epimerization, resulting in the production of secondary BAs [41]. Significant induction of ATP‐binding cassette subfamily G member 5/8 (ABCG5/G8), a bile acid transporter, was observed in polyphenol‐treated hepatic and enterocyte cells [42, 43, 44], but the role and abundance of this family in adipocytes exposed to polyphenols are still poorly described. BAs are better studied by activating G protein‐coupled bile acid receptor 1 (GPBAR1) in the cell membrane, and their expression is regulated by polyphenol agents [45, 46]. GPBAR1 in turn stimulates the production of cAMP, a second messenger involved in many cellular roles [47, 48]. This cAMP rise in concentration is known to activate protein kinase A (PKA), which in turn phosphorylates hormone‐sensitive lipase (HSL) and perilipin 1 (PLIM1), resulting in the breakdown of triglycerides stored in adipose tissue [16, 49]. The cAMP also activates cAMP response element‐binding protein (CREB), which interacts with PGC‐1α to promote browning [50]. Anthocyanins are capable of increase the phosphorylation of CREB through PKA activation, which induces a CREB‐mediated upregulation [51].

### 5.2 Intracellular Signaling From FXR Receptor to PGC1α

The farnesoid X receptor (FXR) has a complex role in brown adipocyte activation, acting as both an agonist and antagonist [25, 40, 52]. FXR agonists, such as fexaramine, have been shown to increase the expression of browning genes in brown adipose tissue (BAT) and gonadal white adipose tissue (gWAT) [53]. Polyphenol supplementation in diabetic obese (db/db) mice can improve glucose metabolism by inhibiting FXR, leading to increased primary bile acid synthesis and decreased secondary bile acid levels, this reduction further weakens FXR action, as they act as FXR agonist [40]. The mechanism by which hydrophobic bile acids enter these cells and bind to FXR remains to be fully clarified, with both active transport and passive diffusion being potential possibilities.

FXR can form a heterodimer with retinoid X receptor α (RXRA), directly binding to PGC1α and modulating its activity [40]. PGC1α sends a signal to PR domain containing 16 (PRDM16), a protein that plays a critical role in differentiating cells into brown fat cells [54]. It works together with CCAAT‐enhancer‐binding protein β (C/EBP‐β), to trigger this switch, as forcing the expression of both PRDM16 and C/EBP‐β in normal skin cells (fibroblasts) is enough to turn them into brown fat cells [19]. PRDM16 is a zinc finger protein type, known to directly participate in DNA binding regions that allow transcription of browning‐related genes [55]. PRDM16‐C/EBPβ, PGC1α, and PGC1β are all possibly repressed by increased FXR expression (Yang et al., 2023b). SMYD1 (histone‐lysine N‐methyltransferase) has recently been associated with either cold‐induced or diet‐induced thermogenesis, demonstrating that SMYD1 is activated in WAT and BAT, working alongside other transcription factors (Cicatiello et al., 2024) [56]. The expression of this protein is higher in beige adipocytes than in white adipocytes, and its silencing leads to impaired mitochondrial activity.

Sirtulin 1 (SIRT1), a nuclear protein, also found in mitocondria [57], interacts with and deacetylates PGC‐1α in an NAD+‐dependent manner, and this interaction influences the expression of gluconeogenic genes mediated by PGC‐1α [58, 59]. However, SIRT1 may not regulate PGC‐1α’s effects on mitochondrial genes [60]. Given its NAD+‐dependent sensing of nutrient fluctuations, SIRT1 likely functions as a key regulator of PGC‐1α‐dependent gene expression [58].

### 5.3 Src Family Kinases Inhibition

Src family kinases (SRCs) are a regulatory brake on developing brown and beige adipose tissue. In humans, it is found to be positively correlated with adipocyte differentiation [61]. Inhibiting SRCs through various approaches has been shown to promote both the differentiation of brown preadipocytes and the browning of white preadipocytes upregulating relevant proteins including PGC‐1α [62]. A notable observation is the decline in SRC activity during the maturation of both brown and white adipocytes, while SRC is expressed at much higher levels in WAT than in BAT [62]. SRCs are activated by ADBR3, and are required to activate extracellular signal‐regulated kinases (ERK1/2) and reach nuclear co‐factors in brown adipocytes [63, 64].

Peroxisome proliferator‐activated receptor gamma (PPARG), another PGC‐1α coactivator, was shown to interact directly with SRC, influencing each other's activity. SRC can suppress PPARG transcriptional activity in a kinase activity‐independent manner through protein–protein interactions [65] and through ERK1/2 [66, 67]. Additionally, SRC interacts with PPARG to reduce lipolysis via fatty acid binding protein 4 (FABP4) [65].

Several polyphenol molecules have been identified as inhibitors of SRC, with potential implications for inflammation and carcinogenesis [68, 69]. Despite SRC's promising role in adipogenesis and thermogenesis regulation, research specifically examining its direct interaction with polyphenols in these contexts is limited.

### 5.4 Estrogen‐Related Receptor Alpha (ERRα)

ERRα is known to be involved in thermogenesis and the selective activation of the protein can induce the begeing of adipocytes [70]. ERRα is long known to physically interact with PGC1α after phosphorylation [71, 72]. Together, they regulate gene expression related to metabolic processes, mitochondrial function, and transcriptional activity [73]. Additionally, ERRα forms a heterodimer with PGC1α that binds to response elements in the ESRRA gene promoter region, leading to an increase in its expression [74].

The estrogen‐Wnt signaling cascade can upregulate the expression of FGF21 in a PPARα and PGC1α‐independent pathway [75]. Many polyphenolic substances, known as phytoestrogens, can interact with ERRα and regulate energetic metabolism [76, 77]. This interaction with female hormones and lipidic/thermogenic pathways may partially explain why males, but not females, exhibit impaired fat tolerance in some studies [37, 78].

### 5.5 Other Transcriptional Co‐Activators

Among other well‐studied proteins that are modulated by polyphenol activity, Nuclear Respiratory Factor 1 (NRF1), Sterol Regulatory Element Binding Protein 1‐c (SREBP‐1c), and mitochondrial transcription factor A (TFAM) are proteins intimately linked to PGC1 coactivators and modulate transcriptional activity of many thermogenic genes [79, 80, 81]. NRF1 is involved with key metabolic genes that regulate nuclear genes required for mitochondrial respiration and mitochondrial DNA transcription and replication [82]. SREBP‐1c is a key protein regulating lipogenesis [83], known to interact directly with SIRT1 [84]. TFAM binds directly in the mtDNA and is involved with replication, repair, and mitochondrial biogenesis [85].

### 5.6 Carnitine Palmitoyltransferases

Carnitine palmitoyltransferase 1A (CPT1A) is an important enzyme found in the outer mitochondrial membrane that plays a vital role in the metabolism of fatty acids, including fatty acid transport, mitochondrial entry, and consequent energy production through carnitine shuttle with carnitine palmitoyltransferase 2 (CPT2) in the inner membrane [86]. PGC1A knock‐out can decrease the transcription levels of both proteins [87] and have their abundance upregulated by polyphenol administration in human cells [88, 89] and mice [90]. Together, they are responsible for facilitating lipid influx into the mitochondria, fueling UCP1‐mediated uncoupling and therefore essential for thermogenesis.

## 6 Phenolic Compounds in Cell Models

While there are many types of phenolic compounds, the majority of tested compounds are known to modulate lipogenesis and obesity to some extent, also promoting mitochondrial biogenesis (Table 1). Quercetin treatment significantly upregulated UCP1, PGC1α, and Tfam expression in 3T3‐L1 adipocytes, suggesting its potential to induce browning in human cells [91]. Quercetin's effect on UCP1 induction appears to be mediated by its interaction with the PPARγ as evidenced by the differential UCP1 expression observed in quercetin‐treated 3T3‐L1 adipocytes in the presence or absence of a PPARγ antagonist [91]. Also with 3T3‐L1 adipocytes, it was observed that *Smilax china* L. extract, engelitin, quercetin, and caffeic acid can turn white fat cells into beige or brown‐like fat cells [92]. Many of the enzymes involved in the regulation of lipolysis (adipose triglyceride lipase [ATGL] and PKA) and β‐oxidation (PPARα, CPT‐1, carboxylic acid oxidase [ACO]) promoting fatty acid breakdown and energy expenditure. Furthermore, the extract and its monomers potently stimulated the browning program by elevating brown‐specific genes and proteins like UCP1, PGC‐1α, and PRDM16, along with beige‐specific markers [92]. A study using blue honeysuckle berry extract noted reduced fat accumulation by lowering levels of proteins involved in fat production and activating an enzyme that burns fat, also increasing markers for beige fat in a dose‐dependant manner, including UCP1, PPARγ, and C/EBPα [93].

**TABLE 1:** Studies with phenolic compounds targeting thermogenesis in cells.

Blue honeysuckle (*Lonicera caerulea*)	Phenolic extract	3T3‐L1 cells	50 and 100 µg/mL	6 days	⇑ ACC, AMPK phosphorylation, Cd137, Tmem26, UCP1 (PX) ∅ TG, LIPE, ATGL ⇓ Lipid accumulation in adipocytes, FAS, SREBP‐1c, PPARγ, C/EBPα (PX)	[93]
Resveratrol	Stilbenoid	hMADS adipocytes	0.1–10 µm/mL	4 days	⇑ UCP1 up to 1 µm (PX, GX), SIRT, CIDEA (GX) ⇓ UCP1 > 1 µm (PX, GX), CIDEA (GX)	[117]
6‐Gingerol	Gingerol	3T3‐L1 preadipocytes	20‐150 µg/mL	6 days	⇑ UCP1, PRDM16, PGC1 α, PPARγ, C/EBP α, AP2, Cidea, Cited1, SIRT1, Tmem26 (GX) ⇓ TG	[94]
*Smilax china* L. engelitin; quercetin; caffeic acid	Phenolic glucoside, flavonoid, phenolic acid	3T3‐L1 adipocytes	40 µg/mL	24 h	⇑ ATGL, UCP‐1, CPT, ACO, PPARα, PGC‐1α, PRDM16 PGC‐1α, PRDM16, Nrf1 and Tfam, Tmem26, Tbx1, CD137, β3‐AR, PKA and pAMPKα (GX and PX). ∅ Cell viability ⇓ Lipid content	[92]

Ethyl acetate extraction of 6‐gingerol, one of the main components of the ginger, is capable of inducing brown adipocyte differentiation of 3T3‐L1 preadipocyte, but not a water extraction of the same root, increasing expression of UCP1, Prdm16, and PGC1α, also doubling NRF1 and TFAM expression [94].

## 7 Thermogenesis Regulation by Phenolic Compounds in Rodent Models

From Table 2, we can observe that UCP1, PCG1α, PRDM16, CIDEA, SIRT1, PPARγ, and CPT1A are the most common upregulated proteins across many tissues upon polyphenol administration. Regarding the study of pure substances, in an study with resveratrol and pterostilbene, it was observed that both can prevent triglyceride accumulation in brown adipose tissue and increased UCP1 expression, with resveratrol also enhancing SIRT3 expression, while neither compound significantly affected PGC‐1α activation in the dose (30 mg/kg) given; these results suggest that their effects on thermogenic capacity depend on metabolic status and the specific conditions of a high‐fat, high‐fructose diet [95]. In another study, pterostilbene administration was observed to significantly increase UCP1 protein levels, despite no observed changes in UCP1 mRNA expression [96]. Mice fed a high‐fat diet supplemented with a dietary achievable dose of protocatechuic acid (0.003%) can experience significant reductions in body weight gain, improve insulin sensitivity, and decrease fat accumulation in the liver [97]. These effects were not linked to increased fat burning in adipose tissue, but rather likely to PCA's enhancement of fat processing within the liver and adipose tissue through increased activity of the CPT1A enzyme [97].

**TABLE 2:** Studies with phenolic compounds targeting thermogenesis in rodents.

Phenolic compound	Class	Model	Daily dose	Time	Finding	Reference
Magnolol, Hinokiol	Lignans	Mouse	30 mg/kg	8 weeks	⇑ PPARα, PPARγ and UCP1, fatty acid oxidation, enhanced expression and secretion of FGF21, tissue browning ⇓ lipogenesis	[100]
Protocatechuic acid	Phenolic acid	Mouse	0.003%	16 weeks	⇑ Insulin sensitivity ∅ No UCP1 change in tissues ⇓ Body wt gain, and attenuated hepatic steatosis.	[97]
Pterostilbene and resveratrol	Stilbenoid	Mouse	30 mg/kg	8 weeks	⇑ UCP1 and SIRT3 (PX) for resveratrol ∅ No change in food intake, iBat wt, SIRT1 expression, ATGL, CD36, FATP1, NRG4, EPDR1, GLUT4, and HSL expression ⇓ BW for resveratrol, triglyceride accumulation in ibat, prevented greater CPT‐1A activity, NRF1 expression for stilbene, FAS expression for both	[95]
Conventional grapes and organic grapes	Polyphenol	Mouse	100 mg/kg	10 weeks (6,12,18 photoperiods)	⇑ UCP1 and leptin expression in BAT, browning of iWAT, energy expenditure ∅ TG, TC	[106]
*Smilax china* L. rhizome	Extract	Mouse	100–400 mg/kg	10 weeks	⇑ GLUT4, IRS1, IRS2, AKT, ACO, PKA (GX); AKT, p‐AKT, GLUT4, UCP‐1, AMPK, and p‐AMPK (PX) in liver ∅ Food Intake ⇓ BW, (SL of) HDL LDL, TC, and TG, GLU, Insulin, IR, IL‐6, MCP‐1, and TNF‐α, fat accumulation; mTORC1, SREBP1c, FAS, HMGCR, FOXO1 (GX); SREBP1c, FAS, HMGCR, p‐IKBα, and P65 (PX) in liver	[99]
Curcumin	Curcuminoid	Mouse	20 mg/ kg	6 weeks	⇑ UCP1, PRDM16 expression in BAT and iWAT (PX), GT ∅ BW, adiposity, food intake, iBAT wt, PGC1‐α (PX) ⇓ TC, TG, glucose, Insulin	[98]
Tetrahydrocurcumin	Curcuminoid	Mouse	20 and 100 mg/kg	14 weeks	⇑ UCP1, Heat production for 20 mg/kg, adiponectin (serum and WAT), GLUT4 in adipocyte, CPT‐1, PPARα (PX) in liver ∅ Liver, kidney, and pancreas, Bat wt ⇓ Blood glucose, spleen wt, TG, TC, food intake for 100 mg/kg, macrophage infiltration and modulation M1/M2 in eWAT, hepatic steatosis, GOT, GTP, ACC, FAS (PX) in liver	[118]
Cocoa extract	flavonol	Mouse	50 mg/kg	2 weeks	⇑ ND, AD excretion, UCP‐1, PGC‐1α, and PRDM16 (GX) in BAT, PGC‐1α, CD137, TMEM26; PRDM16 (GX) in iWAT ∅ UCP‐1 (PX); PRDM16, TMEm26, TBX‐1 (GX) in BAT; UCP‐1, PGC‐1α, CD137, TMEM26, and TBX‐1 (GX) in iWAT	[104]
Blueberry and cranberry extract	Phenolic extract	Mouse	1% and 2% extract	24 weeks	⇑ Fecal SCFAs ∅ Energy intake, MCP‐1 and IL‐1β in plasma ⇓ BW, WAT, Liver WT, TC, TG, LPS, TNF‐α in plasma, SFAs, MUFAs, PUFAs in the liver, F/B ratio in cranberry 2%,	[107]
Quercetin	Flavonoid	C57BL/6 mice	0.05%	9 weeks	⇑ UCP1, PPARγ and PGC1α, TFAM, NRF‐1, PKA, PRDM16, CIDEA, TMEM26 (PX), AMPK phosporylation in WAT, NE in plasma, β3AR (GX) in WAT, UCP1 (PX) in BAT ∅ FI ⇓ WAT wt, BW gain	[91]
Green tea	Phenolic extract	C57BL/6 mice	500 mg/kg	12 weeks	⇑ Insulin sensivity, UCP1, Xbp1s (GX) in BAT, PPARα, CPT1b, Adrb3, Irf4, PPARGC1α, PPARΓ, CIDEA, PDE1B, UCP1, LXR (GX) SWAT ∅ FI, PPARΑ, CPT, ADRB3, IRF4, PPARGC1A, PPARG, CIDEA, DIO2, PDE1B (GX) IN BAT ⇓ BW gain, eWAT and sWAT wt, Glucose, Insulin in plasma, IL‐1β, IL‐6, TNF‐α, IL17, IFN‐γ, and MCP‐1 in plasma, HSPA5 (GX) in BAT and WAT,	[101]
		3T3‐F442A preadipocytes	0.19% concentration	8 days	⇑ Oxygen consumption, PPARα, Adrb3, PPARGC1α, CIDEA, and UCP1 (GX)	
Cranberry polyphenolic extract	Phenolic extract, anthocyanins	C57BL/6J mice	63.8 mg/kg	16 weeks	⇑ Insulin sensivity, VO_2_, EE, ATGL, CPT1A, MCAD (GX) in liver, CPT1A, MCAD, UCP1, PGC1α, CIDEA, Cd137, TBX1 (GX) in iWAT, PPARA, MCAD, UCP1, PGC1α, CIDEA, PRDM16 (GX) in BAT; TMEM26, HOXC8, CD137 in eWAT ∅ FI, Bat wt, VCO_2_, CPT1A, MCAD, PPARA, UCP1 (GX) in eWAT ⇓ BW gain, Liver wt, TNFA, MCP1 (GX) in liver	[102]
Apple	Apple polyphenols	C57BL/6J mice	5% apple polyphenols	4 weeks	⇑ OXPHOS, CD137 (PX) in eWAT; UCP1, CIDEA, TBX1, CD137, FGF21 (GX), UCP1, OXPHOS, PGC1 α, FGF21 (PX), ND in iWAT ∅ Energy intake, UCP1, CIDEA, NDUFB8, SDHB, UQCRC2, COX I, and ATP5A (GX) in BAT; UCP1, CIDEA, TBX1, CD137 (PX) PGC1 α in BAT and eWAT and (GX) in eWAT ⇓ BW gain, BAT, eWAT, iWAT wt,	[103]
		Adipose‐derived stem cells (ASCs)	5–100 µg/mL	4 days	∅ UCP1, PGC‐1α, FGF21 (GX) and (PX) OXPHOS (PX), beige adipocite differentiation. ⇓ Fabp4 (GX)	
Pterostilbene	Stilbenoid	C57BL/6 mice	22, 45, and 90 mg/kg/day	30 weeks	⇑ GT, CIDEA, EBF2, PGC1α, PPARγ, Sirt1, and Tbx1(GX) UCP1 (PX) ∅ CITED1, FGF21, PAT2, PPAR α, UCP1 (GX) ⇓ BW	[96]
		3T3‐L1 preadipocytes	5 µM	1 day	⇑ CIDEA, FGF21 (GX), UCP1 (PX) ∅ CITED1, EBF2, PAT2, PGC‐1α, PRDM16, SIRT1, TBX1, UCP1 (GX) ⇓ PPARγ (GX)	
Resveratrol;	Stilbenoid	C57BL/6 mice	0.5%	12 weeks	⇑ UCP1, PRDM16, SIRT1, PGC‐1α, PPARα, adipo (PX) ∅ mWAT wt, ⇓ BW, LDL, gWAT and rWAT wt,	[119]
Oxyresveratrol			0.1% and 0.5%		⇑ PRDM16, SIRT1, PGC‐1α, C/EBPβ (PX) ∅ MES wt, gWAT wt; UCP1, CPT1, adipo(PX) ⇓ BW, rWat wt;	
Blueberry extract		Mice			⇑ UCP1, PRDM16, PCG1α, PPARα, DIO2, COX7a1, COX8b, CIDEA, Errα (GX) UCP1, PCG1α (PX), ∅ ⇓ TG, LEP,	[25]
Compound 18a	flavonoid	C57BL/6J mice	20–40 mg/kg	9 weeks	⇑ UCP1, PRDM16, PCG1α, TGR5, DIO2 (GX) UCP1, PRDM16, PCG1α (PX), rectal temperature ∅ ⇓ iBAT, iWAT, eWAT wt, BW, GT	[120]

**Abbreviations**: ACC, acetyl‐CoA carboxylase; ACO, acyl‐CoA oxidase; AD, adrenaline; Adipo, adiponectin; Adrb3, adrenoceptor beta 3; AKT, protein kinase B; AMPKα, phosphorylated activated protein kinase alpha subunit; AP2, adaptor protein 2; ATGL, adipose triglyceride lipase; ATP5A, subunit of Complex V; C/EBP α, CCAAT/enhancer binding protein alpha; C/EBPβ, CCAAT/enhancer‐binding protein beta; CD36, cluster of differentiation 36; CD137, cluster of differentiation 137; CIDEA, cell death‐inducing DNA fragmentation factor alpha subunit; COX I, Complex IV; CPT, carnitine palmitoyltransferase 1ª; CPT‐1A, carnitine palmitoyltransferase 1ª; Dio2, Iodothyronine deiodinase 2; EE, energy expenditure; EPDR1, ependymin related Protein 1; FAS, fatty acid synthase; FATP1, fatty acid transport Protein 1; FI, food intake; FOXO1, Forkhead box protein O1; GLUT4, glucose transporter Type 4; GOT, glutamic‐oxaloacetic transaminase; GPT, glutamic‐pyruvate transaminase; GT, glucose tolerance; GX, gene expression; gWAT, gonadal white adipose tissue; HMGCR, 3‐hydroxy‐3‐methylglutaryl‐coenzyme A reductase; HOXC8, homeobox‐containing protein C8; HSL, hormone‐sensitive lipase; HSPA5, heat shock protein Family A (Hsp70) Member 5; iBAT, interscapular brown adipose tissue weight; IL‐1β, interleukin‐1 beta; IR, insulin resistance; Irf4, Interferon regulatory factor 4; IRS1, insulin receptor substrate 1; IRS2, insulin receptor substrate 2; LIPE, hormone‐sensitive lipase; MCAD, medium‐chain acyl‐CoA dehydrogenase; MCP‐1, monocyte chemoattractant Protein‐1; mTORC1, mammalian target of rapamycin complex 1; MUFAs, monounsaturated fatty acids; mWAT, mesentric white adipose tissue; NE, norepinephrine; NRF1, nuclear respiratory Factor 1; NRG4, Neuregulin 4; NDUFB8, subunit of Complex I; Pde1b, phosphodiesterase 1B; PGC‐1α, peroxisome proliferator‐activated receptor gamma coactivator 1 alpha; PKA, protein kinase A; PPARα, peroxisome proliferator‐activated receptor alpha; PPARγ, peroxisome proliferator‐activated receptor gamma; PRDM16, PR domain containing protein 16; PX, protein expression; PUFAs, polyunsaturated fatty acids; rWAT, retroperitoneal white adipose tissue; SDHB, subunit of Complex II; SCFAs, short‐chain fatty acids; sWAT, subcutaneous white adipose tissue; SFAs, saturated fatty acids; SIRT1, Sirtuin 1; SREBP1c, sterol regulatory element‐binding protein 1c; TBX1, T‐box transcription factor 1; TC, total cholesterol; TG, serum triglyceride; Tfam, transcription factor A, mitochondrial; Tmen26, transmembrane protein 26; UCP‐1, uncoupling protein 1; UQCRC2, subunit of Complex III; VCO2, maximum carbon dioxide production; VO2, maximum oxygen consumption; Xbp1s, spliced X‐box binding protein 1; wt, weight.

Curcumin given to pregnant obese mice can improve the adult male offspring's metabolism. Recent research suggests that they gain less weight, have better blood sugar control, and lower cholesterol, which seems linked to increased genes for brown and beige fat burning, the compound might help offspring avoid some negative effects of a mother's high‐fat diet [98]. The curcumin metabolite, tetrahydrocurcumin, testes on diabetic obese mice administered in 20 and 100 mg/kg doses improved hyperglycemia, but the mechanisms differed. The 20 mg/kg upregulated adiponectin signaling (AdipoR1/R2, APPL1) leading to improved insulin sensitivity, glucose metabolism, and beta‐cell function. Additionally, it increased energy expenditure via WAT browning and UCP1 upregulation. The 100 mg/kg dose showed reduced food intake and less expressive positive results, but even better UCP1 expression.

In a dose‐dependent study, *S. china L*. rhizome extract administration increased the expression of AKT, p‐AKT, GLUT4, UCP‐1, AMPK, and p‐AMPK in the liver, and repressed the expressions of SREBP1c [99], suggesting that polyphenols significantly increased PGC‐1α expression, activating it through the insulin receptor substrate/protein kinase B (IRS/AKT), AMPK, and nuclear factor kappa‐light‐chain‐enhancer of activated B cells (NF‐κB) signaling pathways. The effect of *S. china L*. polyphenols on obesity parameters at low (100 mg/kg), medium (200 mg/kg), and high doses (400 mg/kg) significantly reduced body weight gain and intraperitoneal adipose tissue index, with the most pronounced effects observed at the high dose. Additionally, higher doses corresponded to greater reductions in serum lipid levels and inflammatory factors, enhancing insulin sensitivity. Magnolol and Honokiol, compounds found in *Magnolia officinalis*, are reported to function as dual agonists of PPARα and PPARγ receptors, in a dose‐dependent fashion. Experiments in male mice (30 mg /kg for 8 weeks) with both compounds increased the liver's production of FGF21, as well as CPT1A, CPT1B, and CPT2 levels, increasing tissue browning and thermogenesis while reducing the weight of adipose tissues [100].

The treatment with green tea 500 mg/kg gavaged for 12 weeks is capable of effectively reducing circulating fatty acids by promoting their uptake in white adipose tissue and increased energy expenditure, likely contributing to lower liver fat storage [101]. While the precise mechanism for the thermogenic response is unclear, green tea catechins seem to initially influence PPARγ, potentially setting the stage for a more metabolically active fat tissue. Also, phenolic extract from cranberries can upregulate the activity of PRDM16 in BAT, but not in eWAT [102]. Observations made with apple polyphenols suggest that the increased thermogenic capacity induced by intake is the result of iWAT adaptations, not from BAT or eWAT changes [103]. This dissociation between mRNA and protein abundance has been reported by other researchers in different models, suggesting potential post‐transcriptional regulatory mechanisms, such as protein turnover, might be involved in UCP1 regulation.

Adrenergic receptors’ dependence on cocoa flavanol‐induced adipose tissue browning was identified in C57BL/6J mice orally administered cocoa extract [104]. After 2 weeks, a significant increase in norepinephrine and adrenaline excretion and increased expression of browning protein markers (UCP‐1, PGC‐1α, PRDM16) was observed. Specific β‐adrenergic blockers demonstrated that these proteins’ expression increase could be prevented following flavanol administration [105]. While the association between adrenergic receptors and cold‐induced thermogenesis is well‐established, the underlying mechanisms of their interdependence in adipose tissue browning, require further elucidation.

Polyphenol consumption, both in terms of quantity and quality, can affect the body's response to light exposure. A study by Navarro‐Masip et al. [106] evaluated how different photoperiods, simulating seasonal changes, influence body, and protein parameters in Fischer 344 rats fed with common or organic grapes. The rats were exposed to 6, 12, or 18 h of light per day. The study found that energy expenditure decreased in rats exposed to winter‐like light periods (6 h), while it increased in rats exposed to longer light periods (12 and 18 h) [106]. Additionally, the browning of iWAT was observed only in the 12 and 18‐h light periods, raising intriguing questions about evolutionary adaptations for thermogenesis and seasons.

Blueberry and cranberry anthocyanin extracts reduced body weight and fat accumulation in mice fed a high‐fat diet, but only at the higher dose (2%) cranberry juice could reduce body weight [107]. These extracts may work by reducing inflammatory markers and promoting SCFAs release while lowering LPS production by gut microbiota modulation, potentially offering a strategy for managing obesity and its health risks [107].

## 8 Evolutionary Relationship and Perspectives of Thermogenesis and Polyphenols Studies

While it is obvious that cold temperatures can trigger BAT‐enhanced activity and browning of WAT, why the phenolic compounds can mimic this activity in mice and humans is quite intriguing. The first conspicuous example that comes to mind is hibernating bears. Brown bears pack on fat for hibernation by gorging for hours on berries, high in fructose and polyphenols, in summer and fall [108]. The fat‐burning thermogenesis promoted might be linked to how the bear's body handles uric acid, a byproduct of fructose metabolism [109]. Interestingly, it was investigated recently that high‐frutose diet suppresses glucose uptake in human BAT [110].

Several studies support the notion that these compounds activate gene transcription pathways leading to enhanced thermogenic activity, including mitochondrial proliferation and improved lipid metabolism [98, 103, 111]. The evolutionary significance of this interdependence and its implications for these animals is not currently known. Nevertheless, non‐hibernating animal models, such as mice, have been extensively studied for this purpose, demonstrating adipose tissue browning in response to the intake of these compounds. Whether these pathways represent evolutionary vestiges or possess adaptive functions for these individuals and the extent they are relevant in humans also remains to be elucidated. Due to the higher abundance of brown adipose tissue (BAT) in human newborns compared to adults, BAT is widely recognized to play a critical role in thermoregulation in infants, who cannot protect themselves from cold temperatures [112]. A recent study with Scandinavian swimmers has shown increased BAT activity while swimming in cold waters [113].

On the other way, acute fructose administration, but not glucose, rapidly raises FGF21 levels in humans [114, 115]. The mammalian *FGF21* gene is highly conserved, indeed, there is a difference of only one or two amino acids between humans and gorillas or humans and chimpanzees, respectively, and there is nearly 80% homology between human and rodent FGF21s [116]. This protein, highly expressed in the liver, is known to induce the browning of iWAT, through PPARα induction [36], leading to lipolysis. Also, as mentioned, ADBR2 not ADBR3 is the main target for pharmacological activation of human brown adipocytes. How FGF21R interacts with these receptors to propagate thermogenic signals in humans might differ from mice, more responsive to ADBR3, considerably.

While recent articles focus on the relationship between microbiota and FXR as possibly responsible for thermogenic activation by polyphenols, the role of this activation and the differences between these metabolites and structures regarding more effective activation are yet to be determined. The full extent of why consuming these berries, rich in phenolic compounds, may induce browning of adipose tissue in humans, and whether there is any correlation with cold‐stressed animals, is still to be clarified.

## Conflicts of Interest

The authors declare no conflicts of interest.

## Peer Review

The peer review history for this article is available at https://publons.com/publon/10.1002/mnfr.70072.

## Data Availability

No data are available.
